# A Novel Strategy for Efficient Agaro-Oligosaccharide Production Based on the Enzymatic Degradation of Crude Agarose in *Flammeovirga pacifica* WPAGA1

**DOI:** 10.3389/fmicb.2019.01231

**Published:** 2019-06-12

**Authors:** Boliang Gao, Li Li, Hui Wu, Du Zhu, Min Jin, Wu Qu, Runying Zeng

**Affiliations:** ^1^Key Laboratory of Bioprocess Engineering of Jiangxi Province, College of Life Sciences, Jiangxi Science and Technology Normal University, Nanchang, China; ^2^State Key Laboratory Breeding Base of Marine Genetic Resource, Third Institute of Oceanography, SOA, Xiamen, China; ^3^State Key Laboratory of Bioreactor Engineering, East China University of Science and Technology, Shanghai, China; ^4^Fujian Collaborative Innovation Center for Exploitation and Utilization of Marine Biological Resources, Xiamen, China

**Keywords:** *Flammeovirga pacifica* WPAGA1, metabolic pathway, crude agarose, agaro-oligosaccharides, biosynthesis

## Abstract

To avoid conflict between biofuel and food resource production, marine macroalgae (main algal polysaccharides) have been suggested as potent feedstock for biofuel production. *Flammeovirga pacifica* WPAGA1, a typical marine polysaccharide-degrading bacterium, can utilize crude agarose as the sole carbon source. Transcriptomic analysis was performed to further investigate the metabolic pathway of environmental-friendly utilization of crude agarose in *F. pacifica* WPAGA1. All these enzymes were overexpressed in *Escherichia coli* BL21(DE3), and the purified enzymes were characterized *in vitro*. As a result, the pathway of crude agarose which is desulfurized and hydrolyzed by enzymes to produce fermentable sugar is clear. Interestingly, sole neoagarobiose (~450 mg/L) was produced from crude agarose as a feedstock using engineered *E. coli* BL21(DE3). This study firstly reveals the metabolic pathway of crude agarose in strain WPAGA1 and establishes a novel and environmental-friendly strategy for neoagarobiose production using crude agarose as cost-effective and non-food-based feedstock.

## Introduction

Marine macroalgae serve as the critical roles of marine energy, material, and carbon cycles in marine ecosystems. The major carbohydrate compositions of marine macroalgae are typical marine polysaccharides, such as agar, carrageenan, porphyran, ulvan, fucan, and alginate, and they are different from those of terrestrial plants (Goh and Lee, 2010; Hehemann et al., 2012). Some of them have a small amount of lignin in the cell wall and intercellular space. Many previous studies have reported that these polysaccharides have potential applications in food, cosmetic, and medical industries (Zhang et al., 2010; Güner et al., 2015; Wang et al., 2017). In recent years, to avoid conflict between biofuel and food resource production, marine macroalgae (main algal polysaccharides) have been proposed as potential feedstock for biofuel production (Horn et al., 2000; Winterton, 2000; Gross, 2008; Baghel et al., 2015). Algal oligosaccharides with the degree of polymerization between 2 and 20 can be produced naturally or derived from polysaccharides after chemical, physical, or biochemical degradations. Compared with algal polysaccharides, algal oligosaccharides are more valuable because of their better biological activities. In the last decade, algal oligosaccharides have been widely utilized in various fields, such as prebiotics, human biological processes, drug carriers, and plant elicitors (Kobayashi et al., 1997; Jang et al., 2009; Jiao et al., 2011). However, the production of algal oligosaccharides, especially for obtaining stable components, is quite difficult and wasteful. Thus, lots of interests were focused on the algae resources, especially algal polysaccharides and oligosaccharides, along with the increasing exploitation and investigation of marine resources.

The traditional method for producing agar-oligosaccharides by chemical degradation is wasteful, difficult, and harmful to the environment. Compared with chemical treatments, biodegradation is an efficient and clean method for producing agar-oligosaccharides from agarose. Several previous studies have found that a large amount of β-agarases, which mainly belong to GH16 and GH86 family, degrade agarose (Michel et al., 2009) and that the producers of hydrolysis are the main neoagarotetraose (NA4) and neoagarohexaose (NA6) (Chi et al., 2012); however, the metabolism of these lack studies. To effectively utilize agarose in the production of biofuel or other chemicals, it must be converted into fermentable sugars. Meanwhile, most natural agarose are sulfated polysaccharides, and their utilization without further processing (Jiao et al., 2011) and production of agar-oligosaccharides by using agarose are difficult. Accordingly, if an effective bioconversion process for sulfated agarose (crude agar) can be developed, the polysaccharide of red algae (main sulfated agarose) can be used in the production of agar-oligosaccharides or industrial biofuels without desulfurization.

*F. pacifica* WPAGA1, which belongs to the genus *Flammeovirga*, can be isolated from deep-sea sediments and can degrade various complex polysaccharides (Xu et al., 2011; Gao et al., 2017). The other members of this genus, such as *Flammeovirga* sp. MY04 (Han et al., 2012) and *Flammeovirga* sp. SJP92 (Dong et al., 2017), can also degrade polysaccharides, especially agarose. Furthermore, our group used *Gracilaria lemaneiformis* (main crude agarose) powder for *F. pacifica* WPAGA1 degradation to produce agar-oligosaccharides. However, the concentration of agar-oligosaccharide produced using the above-mentioned approach was low because these sugars were utilized by strain WPAGA1. Thus, an effective strategy for producing agar-oligosaccharides in other bacteria that cannot utilize oligosaccharides should be developed. In recent years, the development of synthetic biology and metabolic engineering techniques has provided a great opportunity to synthesize or produce agar-oligosaccharides in microorganisms (Andrianantoandro et al., 2006). Accordingly, investigation of the systematic metabolic pathway of sulfated agarose in *F. pacifica* WPAGA1 is a key step and provides theoretical basis for producing agar-oligosaccharides using synthetic biology. Unfortunately, although previous studies have reported some enzymes (most belonging to β-agarase) from these strains, which can hydrolyze agarose to agar-oligosaccharides, the global understanding of metabolic processes of agarose, especially crude agarose, as well as its degradation, has not yet been elucidated.

In this study, we first reported the global metabolic pathway of sulfated agarose in *F. pacifica* WPAGA1 and established a novel strategy for the production of agar-oligosaccharides. We analyzed these genes involved in sulfated agarose metabolism in *F. pacifica* WPAGA1 by using transcriptome analysis. Then, these enzymes were overexpressed in *E. coli* BL21(DE3), and we examined the enzymatic reactions *in vitro*. The corresponding products were analyzed by thin-layer chromatography (TLC), ion chromatography, and mass spectrometry. Furthermore, these enzymes were co-expressed in *E. coli* BL21(DE3) in different combinations, resulting in production of NA4, NA6, and NA2. This work demonstrates the metabolic pathway of sulfated agarose in *F. pacifica* WPAGA1 and provides a novel and costly method of producing agar-oligosaccharides for the utilization of crude polysaccharide.

## Materials and Methods

### Bacterial Strains and Plasmids

Pure NA2, NA4, and NA6 were obtained from Marineoligo (Qingdao, China) and used as standards. The D-galactose standard was obtained from Sigma. Given that 3,6-anhydro-L-galactose (AHG) is not available commercially, the D-form of AHG, 3,6-anhydro-D-galactose (STANDARDS, Shanghai, China), was used as the standard for AHG. *F. pacifica* WPAGA1 was isolated from deep-sea sediments and characterized in our previous study. *E. coli* DH5α and *E. coli* BL21(DE3) were used as hosts for gene cloning or expression, respectively; and pEASY-Blunt E2, pACYCDuet-1, and pET28a (+) were used to clone or express genes. To produce the corresponding sugars in *E. coli* BL21, the plasmids pACY-AGA1, pACY-NAB1, and pET-Sul1 were constructed (the detailed methods were showed in Supplementary Figure S1 and Supplementary Table S3). The detailed bacterial strains and plasmids used in this study are listed in Table 1.

**Table 1:** Strains and plasmids used in this study.

Name	Characteristics	Source
**Strains**		
*Flammeovirga pacifica* WPAGA1	Polysaccharides-degrading marine strain	Our laboratory
*E. coli* DH5α	The host strain for gene cloning	Our laboratory
*E. coli* BL21(DE3)	The host strain for gene expressing	Our laboratory
**Plasmids**		
pEASY-Blunt E2	Gene expressing, ampicillin resistant	TransGene, China
pEASY-AGA950	Agarase gene Aga950 on pEASY-Blunt E2 vector	This study
pEASY-AGA1950	Agarase gene Aga1950 on pEASY-Blunt E2 vector	This study
pEASY-AGA1957	Agarase gene Aga1957 on pEASY-Blunt E2 vector	This study
pEASY-AGA1974	Agarase gene Aga1974 on pEASY-Blunt E2 vector	This study
pEASY-AGA2050	Agarase gene Aga2050 on pEASY-Blunt E2 vector	This study
pEASY-AGA2593	Agarase gene Aga2593 on pEASY-Blunt E2 vector	This study
pEASY-AGA4007	Agarase gene Aga4007 on pEASY-Blunt E2 vector	This study
pEASY-AGA4591	Agarase gene Aga4591 on pEASY-Blunt E2 vector	This study
pEASY-AGA4779	Agarase gene Aga4779 on pEASY-Blunt E2 vector	This study
pEASY-AGA4974	Agarase gene Aga4974 on pEASY-Blunt E2 vector	This study
pEASY-AGA4975	Agarase gene Aga4975 on pEASY-Blunt E2 vector	This study
pEASY-AGA2660	Agarase gene Aga2660 on pEASY-Blunt E2 vector	This study
pEASY-AHGAD4985	AHG dehydrogenase AHGAD4985 on pEASY-Blunt E2 vector	This study
pEASY-AHGAD4649	AHG dehydrogenase AHGAD4649 on pEASY-Blunt E2 vector	This study
pEASY-AHGAC4986	AHGA cycloisomerase AHGAC4986 on pEASY-Blunt E2 vector	This study
pEASY-NABH4900	Glycoside hydrolase NABH4900 on pEASY-Blunt E2 vector	This study
pEASY-NABH4989	Glycoside hydrolase NABH4989 on pEASY-Blunt E2 vector	This study
pEASY-NABH4454	Glycoside hydrolase NABH4454 on pEASY-Blunt E2 vector	This study
pEASY-NABH4302	Glycoside hydrolase NABH4302 on pEASY-Blunt E2 vector	This study
pEASY-Sul4345	Sulfatase Sul4345 on pEASY-Blunt E2 vector	This study
pEASY-Sul1970	Sulfatase Sul1970 on pEASY-Blunt E2 vector	This study
pEASY-Sul1971	Sulfatase Sul1971 on pEASY-Blunt E2 vector	This study
pACYCDuet-1	Coexpression of two target genes, chloramphenicol resistance	Novagen
pET28a (+)	Gene expressing, Kanamycin resistance	Novagen
pACY-AGA1	Aga2660 gene on pACYCDuet-1 vector	This study
pET-NABH	NABH4454 gene on pET 28a vector	This study
pACY-NAB1	Aga4007 and Aga2660 genes on pACYCDuet-1 vector	This study
pET-Sul1	Sul1971 gene on pET 28a vector	This study

### Media and Culture

2216E media (0.2% yeast extract, 1% peptone, and sea water) and modified 2216E media [0.2% yeast extract, 1% peptone, and 20% (v/v) crude polysaccharide solution] were used for *F. pacifica* WPAGA1 growth. Luria-Bertani (LB) media, SOC media (2% tryptone, 0.5% yeast extract, 0.05% NaCl, 2.5 mM KCl, 10 mM MgCl_2_, and 20 mM glucose), and modified SOC media [2% tryptone, 0.5% yeast extract, 0.05% NaCl, 2.5 mM KCl, 10 mM MgCl_2_, 20 mM glucose, and 20% (v/v) crude polysaccharide solution] were used for *E. coli* growth. Antibiotics were added when necessary (ampicillin 100 μg/ml, chloramphenicol 25 μg/ml, and kanamycin 25 μg/ml). Isopropyl-β-D-thiogalactopyranoside (IPTG) was added, when necessary, to reach a final concentration of 0.1 mM.

### Transcriptome Analysis and Quantitative Real-time PCR Validation

*F. pacifica* WPAGA1 was cultured at 28°C for 12 h in modified 2216E medium and centrifuged at 12,000 × *g* for 10 min. The bacterial cells were then prepared for RNA isolation, and the strain WPAGA1 grown in 2216E medium was used as the control. Total RNA from strain WPAGA1 was isolated using total RNA Extraction Kit (Bioteke, Beijing, China) according to the manufacturer’s recommendations. RNA quality was assessed by electrophoresis on 1% agarose gel using Agilent 2,100 Bioanalyzer (Agilent, Santa Clara, CA). Paired-end (PE) libraries were prepared according to the Illumina PE library preparation protocol (Illumina, San Diego, CA). Afterward, PE libraries were sequenced on an Illumina Hiseq 2500 sequencing system. Transcriptome *de novo* assembly was carried out with the short-read assembly program, Trinity, to generate unigenes, and the assembly process was as described in Grabherr et al. (2011). BLASTX searches (*E* < 10^−5^) were conducted to screen the unigenes against the Nr^1^, Swiss-Prot protein^2^, KEGG pathway^3^, and COG databases^4^. High-scoring alignments were used to determine the unigene sequence direction. Afterward, unigene sequences were aligned to the protein databases using BLASTX (*E* < 10^−5^) and to the nucleotide sequence database Nt (*E* < 10^−5^) using BLASTN to obtain both protein and functional annotations. Based on the annotations in the protein databases, Blast2GO was used to obtain gene ontology (GO) annotations for the aligned unigene sequences, and Web Gene Ontology Annotation Plot was used to establish GO functional classifications for all unigenes. The unigenes were aligned to the COG database to predict and classify possible functions, and the KEGG database was used to obtain pathway annotations (*E* < 10^−5^). RPKM was used to calculate unigene expression levels, thus eliminating the influence of gene length and sequencing level on the estimation of gene expression. Raw sequences were quality-filtered and mapped to the *F. pacifica* WPAGA1 reference genome (accession number JRYR00000000), available from GeneBank under the accession number SRP136069.

To confirm the transcriptome analysis results, the expression levels of the five groups of significantly differentially expressed genes were detected by qRT–PCR. Total RNA was extracted and cDNA was reverse-transcribed from total RNA using PrimeScript^Ru^ First Strand cDNA Synthesis Kit (Takara). The expression levels of Aga4974, NABH4900, NABH4989, AHGAD4985, AHGAD4649, and AHGAC4986 were investigated by qRT–PCR; the primer sequences are listed in Supplementary Table S1. The standard curve method was used to measure the gene expression levels of the samples, and 16 s rRNA (accession number HQ412594) was used as the reference gene to normalize the reaction. PCR amplification was performed using ABI Step-one Plus PCR System (Applied Biosystems), and the PCR system process was as follows: incubation at 95°C for 2 min, followed by 50 cycles of 95°C for 10 s, 60°C for 10 s, and 72°C for 40 s. Melting curve analysis was performed to confirm the specificity of amplification.

### Recombinant Enzyme Expression and Purification

*F. pacifica* WPAGA1 was cultured at 28°C for 12 h in 2216E medium and used for the extraction of genomic DNA using a bacterial DNA kit (OMEGA). The putative agarases Aga950 (genomic locus = Scaffold1:1169527:1172148:+), Aga1950 (genomic locus = Scaffold1:2434037:2436871:+), Aga1957 (genomic locus = Scaffold1:2447654:2450545:+), Aga1974 (genomic locus = Scaffold1:2474925:2477822:−), Aga2050 (genomic locus = Scaffold1:2586781:2588928:−), Aga2593 (genomic locus = Scaffold1:3294980:3298363:−), Aga4007 (genomic locus = Scaffold2:151723:153918:+), Aga4591 (genomic locus = Scaffold2:946538:948010:+), Aga4779 (genomic locus = Scaffold2:1274932:1277757:+), Aga4974 (genomic locus = Scaffold2:1593848:1599886:+), Aga4975 (genomic locus = Scaffold2:1600908:1606775:+), and Aga2660 (genomic locus = Scaffold1:3380557:3382626:−); putative AHG dehydrogenase of AHGAD4985 (genomic locus = Scaffold2:1626678:1628144:+) and AHGAD4649 (genomic locus = Scaffold2:1044358:1045824:+); putative AHGA cycloisomerase AHGAC4986 (genomic locus = Scaffold2:1628228:1629337:+); putative glycoside hydrolases NABH4900 (genomic locus = Scaffold2:1482341:1483549:+), NABH4989 (genomic locus = Scaffold2:1631693:1632925:+), NABH4302 (genomic locus = Scaffold2:576759:577871:+), and NABH4454 (genomic locus = Scaffold2:767323:768795:−); and putative sulfatase Sul1970 (genomic locus = Scaffold1:2466133:2467836:−), Sul4345 (genomic locus = Scaffold2:627404:628852:+), and Sul1971 (genomic locus = Scaffold1:2467814:2469589:−) genes were amplified from the genomic DNA of strain WPAGA1 using the primers listed in Supplementary Table S2. The PCR-amplified DNA fragments were recycled, ligated into the pEASY-Blunt E2 vector (TRANSGENE, China), and transformed into *E. coli* DH5α. The resulting plasmids shown in Table 1 were sequenced by Invitrogen Co. Ltd. China for confirmation. To further express these enzymes, all of the recombinant plasmids were transformed into *E. coli* BL21(DE3).

These recombinant *E. coli* BL21(DE3) cells were cultured at 37°C in SOC medium with 100 μg/ml ampicillin. Afterward, the recombinant cells were induced at 0.6 OD_600_ with IPTG. After further incubation at 16°C for 12 h, the culture was centrifuged at 10,000 × *g* for 10 min, and the pellets were resuspended in 20 mM PBS buffer (pH 7.4) and disrupted by ultrasonication (290 W, 5 s bursts, 5 s pulses for 20 min). The supernatant was collected as crude enzymes by centrifugation at 15,000 × *g* at 4 C for 30 min to investigate enzymatic activities. To further investigate their activities, the crude enzymes were purified with Ni^2+^-NTA resin according to the manufacturer’s protocol (Thermo, USA), and the purifications were examined by SDS-PAGE.

### Enzymatic Assay

The enzymatic activities of sulfatases toward crude polysaccharide (main sulfated agarose) were determined using the BaCl_2_-Gelatin method described by Dodgson (1961). The diluted enzyme (100 μl) was incubated with 500 μl of crude polysaccharide at 30°C for 2 h. Afterward, the supernatant of the reaction solution (400 μl) was mixed with 3.6 ml of 3% TCA (dissolved in 1 M HCl) and 1 ml of 0.5% BaCl_2_-Gelatin (Sangon Biotech, China). After incubating the mixture solution at room temperature for 15 min, the amount of free SO_4_^2−^ produced by the sulfatases was determined at an absorbance of 360 nm.

To screen the activated agarases, the 3,5-dinitrosalicylic acid (DNS) method was used to detect the release of reducing sugar from the crude polysaccharide (Miller, 1959). The standard reaction contained 100 μl of crude enzymatic solution and 500 μl of crude polysaccharide, and the heat-inactivated crude enzymes were used as controls. After incubation at 30°C for 2 h, the reaction was stopped by immersion in boiling water for 10 min. A total of 250 μl reaction solution was mixed with 750 μl DNS reagent, heated for 10 min in boiling water bath, and then cooled. The absorbance of reducing sugar was measured at 540 nm. To further investigate their products, these activated agarases were purified to detect enzymatic abilities. The enzymatic activities of these agarases toward agarose were assayed by analyzing the release of agaro-oligosaccharides by TLC and ion chromatography. Enzyme solution (100 μl) and PBS buffer (500 μl) (pH 7.4) containing 0.2% (m/v) agarose were incubated at 30°C for 2 h, and the supernatants were prepared for TLC and ion chromatography (the method is shown in the Supplementary Section). To study the ability of agarases in degrading sulfated agarose, the sulfatases and agarases were co-incubated with crude polysaccharide. Agarase solution (100 μl), sulfatase solution (100 μl), and PBS buffer (500 μl) (pH 7.4) containing crude polysaccharide were incubated at 30°C for 4 h. Afterward, the DNS method was used for detecting the release of the reducing sugar as described above. The agarase with PBS solution containing 0.2% (m/v) agarose was used as the positive control, and the agarase with crude polysaccharide was used as the negative control.

The reaction mixture for agar-oligosaccharide enzymatic assay consisted of 200 μl PBS buffer (pH 7.4) containing 1 mg/ml NA4 and NA6, 50 μl purified glycoside hydrolases NABH4454 or crude AHG dehydrogenase AHGD4985, and AHGA cycloisomerase AHGAC4986. These different mixtures were incubated at 30°C for 12 h. The reactions were terminated by immersion in boiling water for 10 min. The products were purified with Sephadex G-10 (Amersham Biosciences, Piscataway, NJ) and then analyzed by TLC and MS. These methods are shown in the Supplementary Section.

### Production of Agar-Oligosaccharides in *E. coli* BL21(DE3)

To produce agar-oligosaccharides, pACY-NAB1 or pACY-NAB1 plasmid and pET-Sul1 were transformed into *E. coli* BL21(DE3). The *E. coli* BL21(DE3) with pACY-NAB1 or pACY-NAB1 plasmid and pET-Sul1 were cultured at 24°C for 48 h in 50 ml modified SOC medium (20%, v/v crude polysaccharide or 0.2%, m/v agarose), and 0.1 mM IPTG was added when OD_600_ was 0.6. The cultures were centrifuged at 10,000 × *g* for 10 min, and the supernatant was collected to prepare the products for TLC and ion chromatography. The methods for preparing the supernatants to be analyzed by TLC and ion chromatography are described in Supplementary Method.

### Analytical Methods

Agaro-oligosaccharides, D-galactose, AHG, and 2-keto-3-deoxy-D-galactonate (KDGal) produced by their corresponding enzymatic reactions were purified using Sephadex G-10. Afterward, agaro-oligosaccharides and D-galactose were analyzed using TLC plates and ion chromatography. On the other hand, the agaro-oligosaccharides produced by *E. coli* BL21(DE3) were analyzed by ion chromatography. The TLC plates were developed using *n*-butanol/acetic acid/water (2:1:1, v/v/v) as a solvent system, and the spots were visualized by spraying 10% (v/v) H_2_SO_4_ and heating at 100°C for 10 min. For ion exchange chromatography analysis, the above-purified products (agaro-oligosaccharides) and the mixed oligosaccharide standards (NA2, NA4, and NA6) were analyzed under the same conditions by anion exchange chromatography (DIONEX, Sunnyvale, CA, USA) equipped with a 250 × 4 mm IonPac column (ASII-HC). After the sample was loaded, the column was washed with the mobile phase (100 mM NaOH, 150 mM NaAc) at a flow rate of 0.25 ml/min for 50 min. The liquid chromatography (LC) plot was acquired by plotting the electrical conductivity of the eluent against the retention time. Furthermore, NA2, D-galactose, AHG, and KDGal, which were purified by Sephadex G-10, were analyzed using MS method.

## Results and Discussion

### Identification Genes Involved in Sulfated Agarose Metabolism by Transcriptomic Analysis

Previous studies have reported that agarose can be degraded by agarases to generate small units, as well as oligosaccharides. Almost all the agarases are found in marine microbiology because agarose is a typical marine polysaccharide (Jang et al., 2009). These agarases are either α-agarases or β-agarases based on whether they degrade α- or β-linkages in agarose. The α-agarases belong to the GH96 family, whereas β-agarases belong to the GH16, GH50, GH86, and GH118 families (Jang et al., 2009). Many studies have revealed that the β-agarolytic system is considerably more common in microbiology than in the α-agarolytic system (Jang et al., 2009). In the β-agarolytic pathway, the agarose polymer was first degraded by various β-agarases to produce agaro-oligosaccharides, such as NA4, NA6, and then NA2 is normally generated by using β-agarases (main GH50 family), and many enzymes have been found to perform this process. The resulting NA2 must be further degraded into monosugars to be utilized in microbiological applications. Many scientists have tried to find the enzymes responsible for this reaction; however, few enzymatic reaction experiments have been able to perform the activity of hydrolysis of NA2 in microbiology. Our previous study has reported that one GH16, one GH50, and 11 GH86 β-agarases, especially four predicted GH117 family enzymes, which can perform the ability of NA2 hydrolase (NABH), are harbored in the genome of *F. pacifica* WPAGA1 (Hou et al., 2015; Gao et al., 2017). Furthermore, our group found that strain WPAGA1 can degrade crude agarose. To further investigate and verify the crude agarose metabolic pathway in strain WPAGA1, we performed transcriptomic analysis on strain WPAGA1 grown on crude agarose as a carbon source to analyze and identify the deduced enzymes involved in the metabolism of crude agarose. As shown in Table 2, more than 4,700 unigenes were transcribed during growth in the crude agarose as carbon source, and 3,181 unigenes were differentially expressed, including 2,753 up-regulated genes and 438 down-regulated genes compared with the control. Among these differentially expressed genes, we sought for differentially up-regulated genes, whose enzymatic functions were similar to those of the predicted enzymes: agarase, GH117 glycoside hydrolase, AHGAD gene, AHGAC gene, and sulfatase. The results showed that eight agarase genes, four GH117 glycoside hydrolase genes, two AHGAD genes, and one AHGAC gene were significantly up-regulated compared with the control. Moreover, a large amount of sulfatase genes (70/81) was more significantly up-regulated than the control. The results of the transcriptomic analysis were validated by Q-PCR (Supplementary Figure S2).

**Table 2:** Summary of transcriptome analysis.

Content	Number
Total clean reads of experimental group (bp)	3,449,635,083 ± 1,628,199,178
Total clean reads of control group (bp)	2,281,285,917 ± 56,661,009
Q20 percentage of experimental group (bp)	95.66 ± 0.1%
Q20 percentage of control group (bp)	95.83 ± 0.08%
Number of unigenes experimental group (bp)	4,711 ± 46
Number of unigenes control group (bp)	4,722 ± 6
Up-expressing of unigenes	2,753
Down-expressing of unigenes	438
Up-expressing of sulfatase genes	70/81
Up-expressing of agarase genes	8/13
Up-expressing of GH117 genes	4/4
Up-expressing of AHGAD genes	2/2
Up-expressing of AHGAC genes	1/1

AHGAD, 3,6-Anhydro-L-galactose dehydrogenase; AHGAC, 3,6-Anhydrogalactonate cycloisomerase.

### Identification of the Utilization Pathway of Crude Agarose in *F. pacifica* WPAGA1

#### Agarases Catabolize Agarose to Produce Neoagarotetraose and Neoagarohexaose in *F. pacifica* WPAGA1

**Figure 1:**
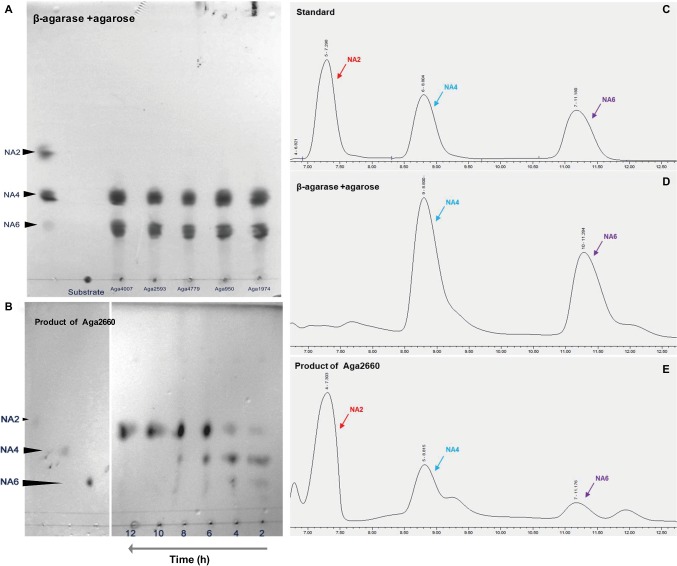
Agarose is hydrolyzed by β-agarases to finally produce neoagarobiose (NA2) in *Flammeovirga pacifica* WPAGA1. **(A)** Analysis of the products of hydrolysis of agarose by Aga4007, Aga2593, Aga4779, Aga950, and Aga1974 using thin-layer chromatography (TLC). **(B)** Analysis of the product of hydrolysis of neoagarotetraose (NA4) and neoagarohexaose (NA6) by Aga2660 by TLC. **(C)** Ion chromatography analysis of the standards: pure NA2, NA4, and NA6. **(D)** Ion chromatography analysis of products of the hydrolysis of agarose by Aga4007, Aga2593, Aga4779, Aga950, and Aga1974. **(E)** Ion chromatography analysis of the products of hydrolysis of NA4 and NA6 by Aga2660.

**Figure 2:**
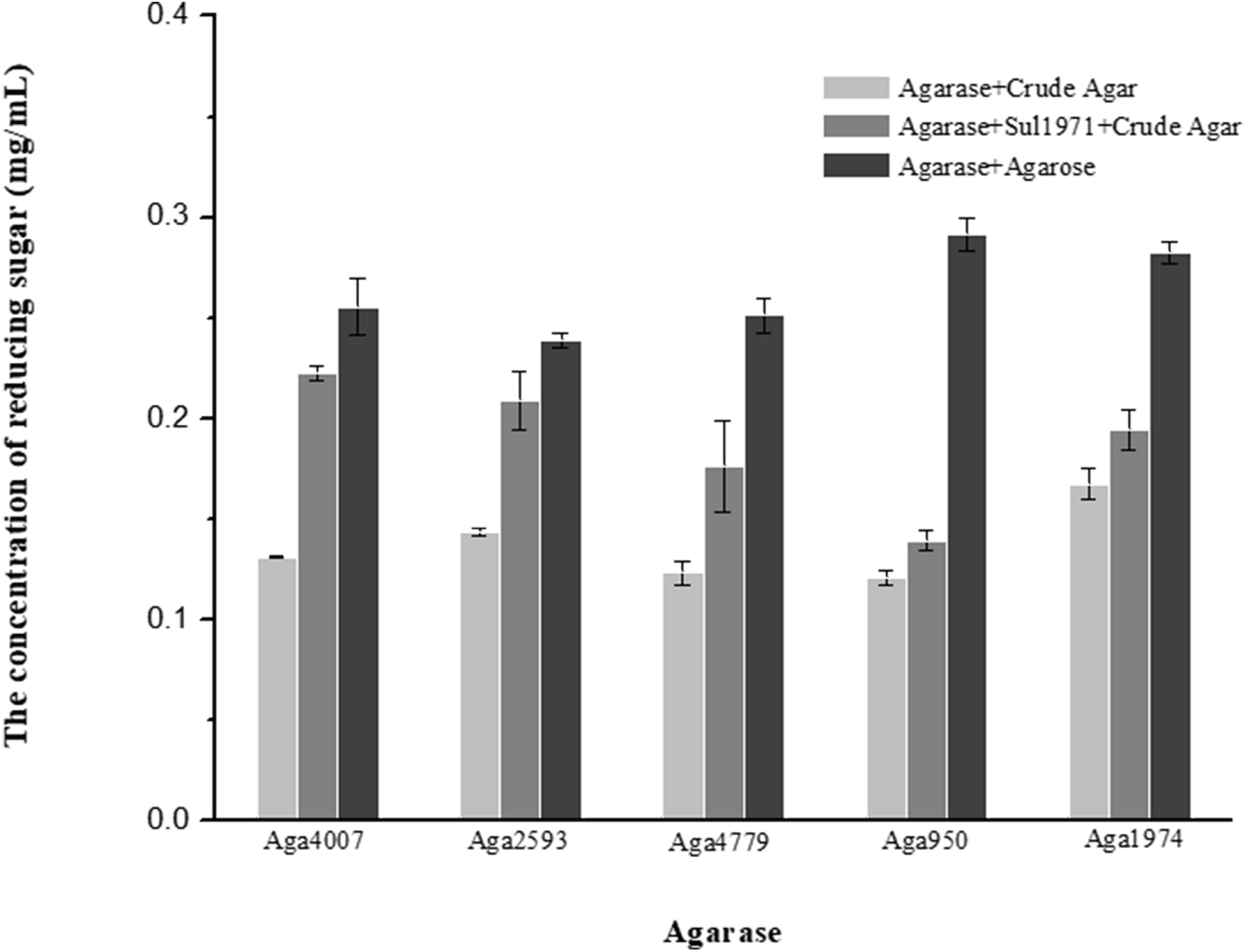
Sulfatase plays a key role in the hydrolysis of crude agarose (sulfated agarose) by agarase. Reducing sugar was produced by agarose hydrolysis of crude agar (gray column), agarases and Sul1971 co-hydrolysis of crude agar (dark gray), and agarase hydrolysis of agarose (black column) (*p* < 0.05).

All 13 predicted agarase genes (as shown above) were cloned and overexpressed in *E. coli* BL21(DE3). To screen the activated agarases, we detected the reducing sugar that was produced by crude recombinant enzymes from agarose using the DNS method. With agarose as substrate, only Aga4007, Aga2593, Aga4779, Aga950, and Aga1974 exhibited catalytic activity for agarose; moreover, Aga4007, Aga2593, Aga4779, Aga950, and Aga1974 produced 0.2554 (after 4 h), 0.2386, 0.2510, 0.2916, and 0.2823 mg/ml reducing sugar, respectively (Figure 2). To further investigate the products of hydrolysis of agarose by agarases, the enzymatic reactions of five purified recombinant agarases were examined *in vitro*. Afterward, the products of these predicted five agarases were analyzed by TLC. As shown in Figure 1A, the products of hydrolysis of agarose by the five agarases are NA4 and NA6. We further analyzed these products by ion chromatography, and the results showed that NA4 and NA6 were the products of the hydrolysis of agarose by these agarases (Figures 1C,D).

#### Sulfatases Play a Key Role in Hydrolysis of Crude Agarose in *F. pacifica* WPAGA1

In our previous study, abundant sulfatase genes (81) were found in the *F. pacifica* WPAGA1 genome (Gao et al., 2017). Most of the sulfatase genes were up-regulated as indicated by transcriptomic analysis, which showed the key step for the metabolism crude agarose by strain WPAGA1. To investigate the role of sulfatase in the crude agarose degradation of strain WPAGA1, we examined the enzymatic reaction of sulfatases *in vitro*. First, the three highest up-regulated sulfatases, as well as Sul1970, Sul1971, and Sul4345, were selected for analysis. Then, three recombinant sulfatases were expressed in *E. coli* BL21(DE3), and the enzymatic activities were detected by the BaCl_2_-Gelatin method. As shown in Supplementary Figure S3, Sul1971 and Sul4345 can produce 0.5264 and 0.1611 mg/ml free SO_4_^2−^ from crude agarose at 37°C for 4 h, whereas Sul1970 was not detected. To further investigate the effect on agarase in its degradation of crude agarose, the Sul1971 and agarases were co-incubated with crude polysaccharide. As shown in Figure 2, Aga4007 can produce 0.2554 mg/ml reducing sugar from agarose after 4 h, whereas 0.1309 mg/ml reducing sugar was produced from crude agarose, and 0.2224 mg/ml reducing sugar was produced from crude agarose with Sul1971, which showed that sulfatase can promote agarase to degrade sulfated agarose. We obtained similar results in other agarases, in which more reducing sugars were detected with Sul1971 than without Sul1971. These results showed that crude agarose was desulfurized by sulfatase and hydrolyzed by agarase to produce NA4 and NA6.

In the marine environment, many polysaccharides, including agarose, exist as sulfated polysaccharides (Witvrouw and De, 1997; Jiao et al., 2011; Wijesinghe and Jeon, 2012). Although almost all of the found metabolic pathway of agarose-degrading in some microorganisms (such as *Agarivorans gilvus* WH0801 or *Postechiella marina* M091) belong to the β-agarolytic system, and the pathway which degrade agarose to generate D-galactose and AHG is clear, but how microbiology utilizing crude agarose is unknown. Thus, marine microorganisms should have an efficient strategy for the utilization of sulfated polysaccharides. As shown in Supplementary Table S4, four polysaccharide-degrading marine bacteria, namely, *Flammeovirga* sp. MY04, *Flammeovirga* sp. OC4, *Flammeovirga* sp. SJP92, and *F. pacifica* WPAGA1, harbor rich genes that encode sulfatase in the genome. Thus, we predicated that these sulfatases play key roles in the initial degradation of crude agarose in marine microorganisms. As a result, significantly, more reducing sugar was produced from crude agarose by the hydrolysis of β-agarases when sulfatase was present (Figure 2). Furthermore, many free SO_4_^2−^ ions were generated from crude agarose by catalyzing sulfatases (Supplementary Figure S3). Thus, it can be predicated that crude agarose is firstly desulfurizing by sulfatase to generate agarose before degrading by agarases.

#### GH50 Family ß-Agarase Hydrolyze Neoagarotetraose and Neoagarohexaose to Generate Neoagarobiose in *F. pacifica* WPAGA1

To investigate the product of the hydrolysis of NA4 and NA6, a GH50 family β-agarase Aga2660 was cloned and overexpressed in *E. coli* BL21(DE3). The enzymatic activity of purified Aga2660 was examined *in vitro* using NA4 and NA6 as substrate. From the enzymatic reaction of Aga2660 *in vitro*, decreases in the amounts of NA4 and NA6, as well as an increase in NA2, were observed by TLC analysis (Figure 1B), and NA2 was detected by ion chromatography (Figure 1E). These results indicated that agarose was first hydrolyzed by GH16 or GH86 family agarase to generate NA4 and NA6; then, NA4 and NA6 were hydrolyzed by GH50 agarase Aga2660 to produce NA2 in strain WPAGA1.

#### Neoagarobiose Is Hydrolyzed by GH117 Family Glycoside Hydrolases to Produce D-Galactose and 3,6-Anhydro-L-Galactose in *F. pacifica* WPAGA1

**Figure 3:**
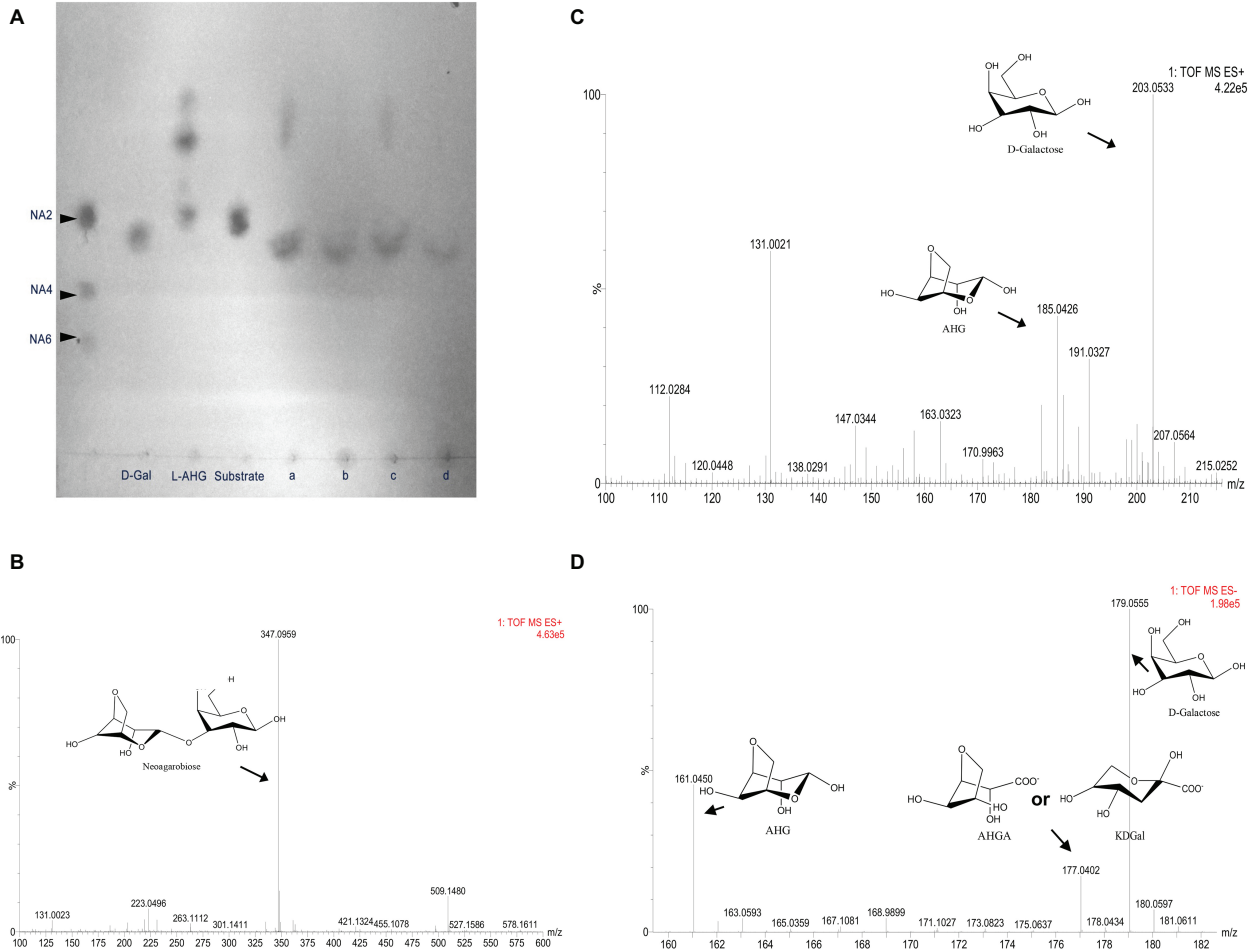
Analysis of the metabolic pathways of NA2 and AHG in *Flammeovirga pacifica* WPAGA1. **(A)** TLC analysis of enzymatic reactions of NABH445, AHGAD4985, and AHGAC4986: NA2 was hydrolyzed by NABH4454 to generate D-galactose and 3,6-anhydro-L-galactose (AHG), with NA2 as a substrate, D-galactose was produced, and AHG was catalyzed by NABH4454 and AHGAD4985, while AHG is not catalyzed when NABH4454 and AHGAC4986 were present, and AHG was also catalyzed when NABH445, AHGAD4985, and AHGAC4986 were present. **(B)** MS analysis of NA2 showed a mass of 347, which is the mass of NA2 (342) plus the mass of Na (23) in positive ion mode. **(C)** MS analysis of the products from the hydrolysis of NA2 by NABH4454, the mass of D-galactose (180) is 203, and the mass of AHG (162) is 185 in positive ion mode (Na^+^). **(D)** MS analysis of the products from catalysis of NA2 by NABH4454, AHGAD4985 or NABH4454, AHGAD4985, and AHGAC4986, the mass of AHGA or KDGal is 177 in negative ion mode.

To analyze and validate the metabolism of NA2, we cloned and overexpressed four putative GH117 glycoside hydrolases, as well as NABH4900, NABH4989, NABH4302, and NABH4454, in *E. coli* BL21(DE3). The four crude recombinant enzymes were examined *in vitro* for the analysis of enzymatic activity using NA2 as a substrate. Our results showed that only NABH4454 can hydrolyze NA2, whereas NABH4900, NABH4989, and NABH4302 were not detected. As shown in Figure 3A, NA2 was hydrolyzed by NABH4454 to produce D-galactose and 3,6-anhydro-L-galactose (AHG). To further validate the molecular function of NABH4454, MS method was performed. For NA2 as a substrate (Figure 3B), we determined the masses of the products, in which D-galactose (mass as 180, m/z + [NA2 + Na]^+^ as 203.0533) and AHG (mass 162, m/z + [AHG + Na]^+^ as 185.0426) were observed after the enzymatic reaction by MS analysis (Figure 3C).

In this study, when strain WPAGA1 was grown on crude agarose as carbon source, the predicate genes (FlaGM004262, FlaGM004568, FlaGM004894, and FlaGM004987) encoding galactose 1-epimerase, which is the key enzyme for Leloir pathway (Ley and Doudoroff, 1957; Bissett and Anderson, 1974; Holden, 2003), were found to be highly expressed (approximately fivefold) than those grown on other carbon sources, as analyzed by transcriptomic analysis. These findings indicated that more galactose might be produced by strain WPAGA1 grown on crude agarose than that grown on other carbon sources and that galactose would then be utilized through the Leloir pathway. Another monosugar, AHG, theoretically comprises 50% molar ratio of the hydrolysis of agarose; thus, AHG should be metabolized in strain WPAGA1 (Suzuki et al., 2002; Ariga et al., 2014; Yun et al., 2017).

#### 3,6-Anhydro-L-Galactose Catalyzed by 3,6-Anhydro-L-Galactose Dehydrogenase and 3,6-Anhydrogalactonate Cycloisomerase to Produce 2-Keto-3-Deoxy-D-Galactonate

AHG is a rare sugar that exists in nature as a monomeric constituent of agarose and has seldom been studied. However, two key enzymes, as well as AHG dehydrogenase and 3,6-anhydrogalactonate (AHGA) cycloisomerase, were recently found in *Vibrio* sp. EJY3, revealing the metabolic pathway of AHG in bacteria (Yun et al., 2015). As shown in our previous study, we found three genes that exhibited high amino sequence similarity to the two key enzymes in the strain WPAGA1 genome. To investigate the metabolic pathway of AHG, two deduced AHG dehydrogenases, AHGAD4985 and AHGAD4649, as well as one deduced AHGA cycloisomerase, AHGAC4986, were cloned and overexpressed in *E. coli* BL21(DE3). NADP^+^ is necessary for the enzymatic reaction of AHG dehydrogenases; therefore, crude enzymes were used for these enzymatic reactions. As shown in Figure 3A, for NA2 as substrate, D-galactose was produced and AHG was catalyzed by the co-incubation of NABH4454 with AHGAD4985 (the activity of AHGAD4649 was not detected), whereas D-galactose was observed and AHG was not catalyzed by NABH4454 and co-incubation of AHGAC4986 with NA2. Afterward, AHG was catalyzed by the enzymatic reaction of NABH4454 and co-incubation of AHGAD4985 with AHGAC4986. To further analyze the products of these enzymatic reactions, the mixed products were purified by Sephadex G-10 and then analyzed using MS method. As shown in Figure 3D, the mass of AHGA was similar to that of KDGal (mass as 178); thus, one m/z + [NA2-H]^−^ mass of 177.0402 was observed.

The metabolic pathway of AHG has been recently studied by two key enzymes, as well as AHG dehydrogenase and AHG cycloisomerase (Yun et al., 2015). Moreover, we found three genes that have high amino similarity with the two enzymes in strain WPAGA1 genome. In this work, we validated the enzymatic activities of the corresponding predicating enzymes (Figure 3). Meanwhile, the spots of AHGA and KDGal were not visualized in TLC analysis. This finding may be attributed to the fact that AHGA and KDGal were mixed with D-galactose or the TLC system was not suitable for AHGA and KDGal. However, the mass values of AHGA and KDGal were detected by MS analysis. Thus, AHG was catalyzed by AHG dehydrogenase and AHG cycloisomerase to generate KDGa, entered the De Ley-Doudoroff pathway and was finally metabolized by glycolysis.

**Figure 4:**
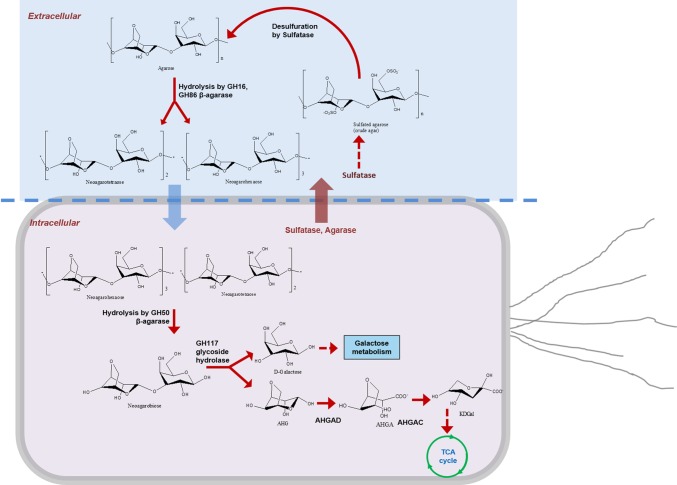
Metabolic pathway of crude agarose in *Flammeovirga pacifica* WPAGA1. Sulfatases and β-agarases (main GH86 and GH16 family) were transported into the bacterial surface; sulfated agarose was desulfurized by sulfatase to produce agarose and hydrolyzed to generate NA4 and NA6; then NA4 and NA6 entered into the bacterial cell and were hydrolyzed by GH50 family β-agarase Aga2660 to produce NA2, and D-galactose and AHG were produced by hydrolysis of NA2 using GH117 family glycoside hydrolase. Finally, D-galactose entered into the galactose metabolic pathway, and AHG was catalyzed by AHG dehydrogenase and AHGA cycloisomerase to form KDGal, which was finally utilized by the TCA cycle.

As a result, we confirmed the metabolic pathway of sulfated agarose (crude agarose) in *F. pacifica* WPAGA1 (Figure 4). First, sulfatases and β-agarases (main GH86 and GH16 family) were transported into the bacterial surface. Sulfated agarose was desulfurized by sulfatase to produce agarose and then hydrolyzed to generate NA4 and NA6, which entered the bacterial cell and were hydrolyzed by GH50 family β-agarase Aga2660 to produce NA2. D-galactose and AHG were produced by the hydrolysis of NA2 using GH117 family glycoside hydrolase. Finally, D-galactose entered the galactose metabolic pathway, and AHG was catalyzed by AHG dehydrogenase and AHGA cycloisomerase to form KDGal, which was finally utilized by the TCA cycle. Thus, our results revealed the enzymatic mechanism of crude agarose as the sole carbon source for *F. pacifica* WPAGA1.

### Production of Neoagarobiose in Recombinant *E. coli* Strains

**Figure 5:**
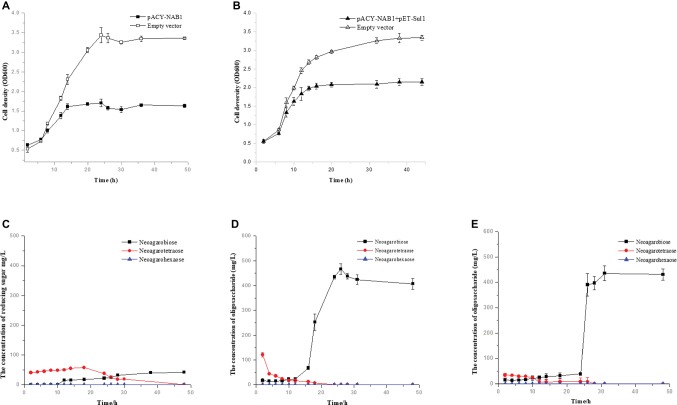
Production of agaro-saccharides using engineered *E. coli*. **(A)** Growth curve of engineered *E. coli* BL21(DE3) harboring pACY-NAB1 or empty vector pACYCDuet-1. **(B)** Growth curve of engineered *E. coli* BL21(DE3) harboring pACY-NAB1 and pET-Sul1 or empty vector pACYCDuet-1 and pET28a (+). **(C)** NA2 production by engineered *E. coli* BL21(DE3) harboring pACY-NAB1 using crude agarose as a feedstock. **(D)** NA2 production by engineered *E. coli* BL21(DE3) harboring pACY-NAB1 using agarose as a feedstock. **(E)** NA2 production by engineered *E. coli* BL21(DE3) harboring pACY-NAB1and pET-Sul1 using crude agarose as a feedstock.

Agar-oligosaccharides are utilized by strain WPAGA1; thus, the latter is unsuitable for producing agar-oligosaccharides. To avoid this question, agar-oligosaccharides were produced using engineered *E. coli* BL21(DE3). To generate a sole agar-oligosaccharide NA2 from agarose, the high-activity agaroses Aga4007 (Figure 2) and Aga2660 were cloned in plasmid pACYCDuet-1 to construct plasmid pACY-NAB1. The plasmid pACY-NAB1 was transformed into *E. coli* BL21(DE3) and grown in SOC medium, which contained 0.2% agarose. We detected the growth profiles of *E. coli* BL21(DE3) strain harboring plasmid pACY-NAB1 and the control strain containing empty vector. The result indicated that the engineered strain entered the stationary phase of growth after 14 h, whereas the control strain entered the stationary phase after 24 h (Figure 5A). Meanwhile, the cell density of the control strain was approximately twofold higher than the engineered strain containing plasmid pACY-NAB1 after 24 h of fermentation (Figure 5A). Furthermore, we detected the concentrations of NA2, NA4, and NA6 in the fermentation of engineered *E. coli* BL21(DE3) strain and control strain. As shown in Figure 5C, the concentrations of NA2, NA4, and NA6 were low based on which crude agarose was used as substrate for the engineered *E. coli* BL21(DE3) strain. With fermentation time, decreases in NA4 and NA6 concentrations, as well as an increase of NA2 concentration, were detected by ion chromatography, and only NA2 was detected after 48 h of fermentation. Agarose was used as substrate for engineered *E. coli* BL21(DE3) strain harboring plasmid pACY-NAB1. Decreases in NA4 and NA6, as well as an increase of NA2, were detected; however, a significantly higher concentration (approximately 500 mg/L) of NA2 was obtained than that in which crude agarose was used as substrate (Figure 5D). To efficiently produce NA2 using crude agarose, plasmid pACY-NAB1 and pET-Sul1 were co-transformed into *E. coli* BL21(DE3). As shown in Figure 5B, the engineered strain harboring plasmid pACY-NAB1 and pET-Sul1 entered the stationary phase of growth after 16 h, and the control strain containing empty vector entered the stationary phase of growth after 30 h, in which the cell density was approximately 1.7-fold that of the engineered strain harboring plasmid pACY-NAB1 and pET-Sul1. Moreover, we analyzed the concentrations of NA4, NA6, and NA2 by ion chromatography. The results showed that NA4 and NA6 concentrations decreased, and that of NA2 increased with fermentation time (Figure 5E). After 30 h of fermentation, only NA2 was produced, and its concentration reached approximately 450 mg/L (Figure 5E), which was almost the same as that in the fermentation of the engineered strain using agarose.

The agaro-oligosaccharides were usually produced *via* chemical methods in the past. These methods, however, were inefficient and unclean strategies for the production of oligosaccharides. Thus, in the present work, our group found a marine polysaccharide-degrading bacterium, *F. pacifica* WPAGA1, which was used to produce agaro-oligosaccharides by fermenting strain WPAGA1, but these oligosaccharides would be utilized by strain WPAGA1. Biosynthesis has been recently used for the production of many compounds that are difficult to obtain using other strategies. Examples are γ-aminobutyric acid and N-acetylneuraminate, which are produced by using engineered bacteria (Andrianantoandro et al., 2006; Wang et al., 2015; Soma et al., 2017; Yang et al., 2017). Using this method, the sole agaro-oligosaccharide, NA2, was produced from crude agarose by *E. coli*. However, its concentration was not high enough to be used in industrial production. The insufficient level of enzymes transferred into the extracellular to hydrolyze crude agarose was the main reason for the low concentration of NA2 in this study. Thus, in our next investigation, we should optimize this strategy for improving the NA2 concentration that is necessary for industrial production. Therefore, we established a novel, clean, and efficient strategy for producing agaro-oligosaccharide using crude agarose as a feedstock.

## Conclusions

As shown in Supplementary Table S4, genes encoding β-agarase, NABH, AHGAD, and AHGAC are found in *Flammeovirga* sp. MY04, *Flammeovirga* sp. OC4, and *Flammeovirga* sp. SJP92 genome, especially genes that encode sulfatase. Thus, these bacteria belonging to genus *Flammeovirga* can metabolize crude agarose and may play a key role in the marine carbon cycle. In conclusion, we have successfully investigated the metabolic pathway of crude agarose in *F. pacifica* WPAGA1, and we have established a novel strategy for producing agaro-oligosaccharide using crude agarose as a feedstock.

## Data Availability

The datasets analyzed for this study can be found in Genebank, accession numbers JRYR00000000, SRP136069 and HQ412594.

## Author Contributions

BG, WQ, and MJ performed the formal analysis. RZ contributed to funding acquisition. BG and LL investigated the data. BG and RZ provided methodology for the study. RZ contributed to project administration. BG, LL, and RZ contributed to resources. RZ supervised the study. BG, WQ, and LL contributed to the validation of data. BG, MJ, HW, and RZ contributed to visualization. RZ contributed to writing – original draft preparation. BG, MJ, HW, DZ, and RZ contributed to writing – review and editing.

### Conflict of Interest Statement

The authors declare that the research was conducted in the absence of any commercial or financial relationships that could be construed as a potential conflict of interest.

## Footnotes

^1^http://www.ncbi.nlm.nih.gov/

^2^http://www.expasy.ch/sprot/

^3^http://www.genome.jp/kegg/

^4^http://www.ncbi.nlm.nih.gov/cog/
