# Supplementary material for: A Novel Strategy for Efficient Agaro-Oligosaccharide Production Based on the Enzymatic Degradation of Crude Agarose in Flammeovirga pacifica WPAGA1
Source: Front Microbiol. 2019 Jun 12;10:1231. doi: 10.3389/fmicb.2019.01231 (PMC6581685; doi:10.3389/fmicb.2019.01231)
Supplement: Supplementary file 1 [file Data_Sheet_1.docx]

| Primer ID | Sequence: 5′→3′ |
| --- | --- |
| 4649Q-F | TGGTGACGCAAGAACTGACG |
| 4649Q-F | ACCACCTTGTTCAACGGCAC |
| 4900Q-F | CCAAAACCAAACGTTGGTTG |
| 4900Q-R | CCACTCGCTTCCATAAACCC |
| 4974Q-F | AAATTAGGTGCTCGTGCTCC |
| 4974Q-R | TGCAGGTACACCCCATGATA |
| 4985Q-F | TTGGCCTTGCAGCTATGGTA |
| 4985Q-R | ACCGTTGTGGAAACCTTGGT |
| 4986Q-F | GAGAAAGCGATTGAATGTGC |
| 4986Q-R | TCAGTGATTTCGGCATAACC |
| 4989Q-F | AGACGTGACCCTTCTGCTTT |
| 4989Q-R | CAAATATCACAACGGTCCCA |

**Supplementary information**

**Table S1.** Primers used in Q-PCR.

**Table S2.** Primers of gene clone used in this study

| Primer ID | Sequence: 5′→3′ |
| --- | --- |
| 1950-F | TCATCATATGCAAGGAAATGCACAAAC |
| 1950-R | CGACAAGCTTTTATTTCTTTATAATCTTATG |
| 1957-F | AATTACTACTATTCAAAGAGACCAGC |
| 1957-R | TTGTATCATAATCTTTTTTATAACTTGATC |
| 1974-F | CGACAAGCTTTTATTCTTTGATAATCCTCTG |
| 1974-R | CGACAAGCTTTTATTCTTTGATAATCCTCTG |
| 2050-F | TCTTTTGGCCAAGATACTTTGGAC |
| 2050-R | TTTTCTAACTATTTTTCTTGATACAATCC |
| 2593-F | AAAACACTTCAACTATTTGTGATTTCGGC |
| 2593-R | TCTAATACTTATTTTCTTTCTAATATCTATTCCATTACC |
| 4007-F | CATCATCATATGCAAAACATTATTGATGTAAAC |
| 4007-R | GTCGACAAGCTTTTAATTAATCAATAATTTTT |
| 4591-F | TATACATATGGGACAAATTGCTAACGC |
| 4591-R | GTGGTGCTCGAGACGGATGATCAGTTTTTG |
| 4779-F | TCATCATATGCAAGATTGGAGTAGTATTCC |
| 4779-R | CGACAAGCTTTTATTTTACTAATATTCTCGC |
| 4974-F | GCGGAGCTCTTAACTTAAATATCGTGCACTCTGT |
| 4974-R | CGGCTCGAGGTTTTTTTACTGAAATTCTTTCTTTCAA |
| 4975-F | GCGGAGCTCGATGTTGATGTTGATGTCAACGT |
| 4975-R | CCGCTCGAGATTTAATAACAATCTTGATTTTATGATCTGC |
| 2660-F | AGTGGAATTGTGTTTATCGCCCTAAAAC |
| 2660-R | GTTATTCACTTTGCCCAATCTATAATCATATAATTGGT |
| 4985-F | TCAGAAACATTAGCAGCAGCA |
| 4985-R | GTATTTCACGTAGAATGTCTTCTTATG |
| 4986-F | AGTACAGCAATTAAAGATATAAAAACAAAGTTATTCG |
| 4986-R | AGCATACTCCTGAGGTTTTACCTCA |
| 4649-F | ACAGATTTTGCAACATCAGTTGGAACA |
| 4649-R | GTTTTGTTGGATGTACATTACTTTAGTTTGTAAGTAC |
| 4900-F | CTAAAAGAACAGCCTGAAAACTCCACT |
| 4900-R | TTTACTTTCTACAACAGATTGATTTTCTTTTT |
| 4989-F | AGAGCAATTAAAATATTAGCAGGAACCGT |
| 4989-R | TCTTGTTGCTTTCGGCTGTTTAGC |
| 4454-F | GCTCAGAATAAGAAGACCTCGAGTACA |
| 4454-R | GTTTTTTAGAAAACTAGATTTATAGAACTCATTCGT |
| 4302-F | CATCATTTTTCAAAACTAACACTAAAGGTTATC |
| 4302-R | TTTTGCATCAAAAGATACAATGGGTATTTG |
| 4345-F | AATAAAACAACTATTATTAAAGTAATTTTACTTTGTGCG |
| 4345-R | TAATTTAAGATTAGTTACCCCTTTTGTTTTATCTCC |
| 1971-F | AGATACTTATCAATCTTCCTTCTTCTAATTTTGGG |
| 1971-R | AATCTTTTCAATATAAACATAGTATGCACCCAC |
| 1970-F | TTTATATTGAAAAGATTTAATGAAATGAAGAAAAACATC |
| 1970-R | ATCGTGGATAAGTACTGGGTGATT |

**Table S3.** Primers used in biosynthesis.

| Primer ID | Sequence: 5′→3′ |
| --- | --- |
| PelB1 | ATCCCATGGCAATGAAATACCTGCTGCCGACCGCTGCTGCTGGTCTGCTGCTCCTCGCTGCCCAGCCGGCGATGGCCCATCACCATCATCACCAC |
| PelB2 | ATCCATATGAAATACCTGCTGCCGACCGCTGCTGCTGGTCTGCTGCTCCTCGCTGCCCAGCCGGCGATGGCC |
| PelB3 | GGCCATCGCCGGCTGGGCAGCGAGGAGCAGCAGACCAGCAGCAGCGGTCGGCAGCAGGTATTTCATTGCCATGGGAT |
| PelB1-F | ATCCCATGGCAATGAAATACCTGC |
| PelB2-F | ATCCATATGAAATACCTGCTGCCG |
| PelB3-R | ATCCCATGGCAATGAAATACCTGC |
| 2660S-F | GGCGATGGCCAGTGGAATTGTGTTTAT |
| 2660S-R | ATCCTCGAGGTTATTCACTTTGCCCAATC |
| 4007S-F | TCATCACCACAAACGAATTTATTTACTACTT |
| 4007S-R | ATAGCGGCCGCATTAATCAATAATTTTTTTGTTT |
| 1971S-F | ATCGGATCCAATCTTTTCAATATAAACATAGT |
| 1971S-R | GGCCATCGCCATGAGATACTTATCA |

**Table S4.** Key genes involved in the metabolism of crude agarose in *Flammeovirga* bacterium.

| Strain | Sulfatase | β-agarase | NABH | AHGAD | AHGAC |
| --- | --- | --- | --- | --- | --- |
| *Flammeovirga* sp*.* MY04 | 74 | 11 | 6 | 1 | 1 |
| *Flammeovirga* sp*.* OC4 | 99 | 5 | 4 | 2 | 1 |
| *Flammeovirga* sp*.* SJP92 | 89 | 7 | 5 | 2 | 1 |
| *Flammeovirga* *pacifica* WPAGA1 | 81 | 13 | 4 | 2 | 1 |

Strain MY04 accession number: NZ_CP003560; Strain OC4 accession number: NZ_JTAM01000001; Strain SJP92 accession number: NZ_LQAQ01000093; Strain WPAGA1 accession number: JRYR00000000. NABH: GH117 family glycoside hydrolase; AHGAD: AHG dehydrogenase; AHGAC: AHGA cycloisomerase.

**
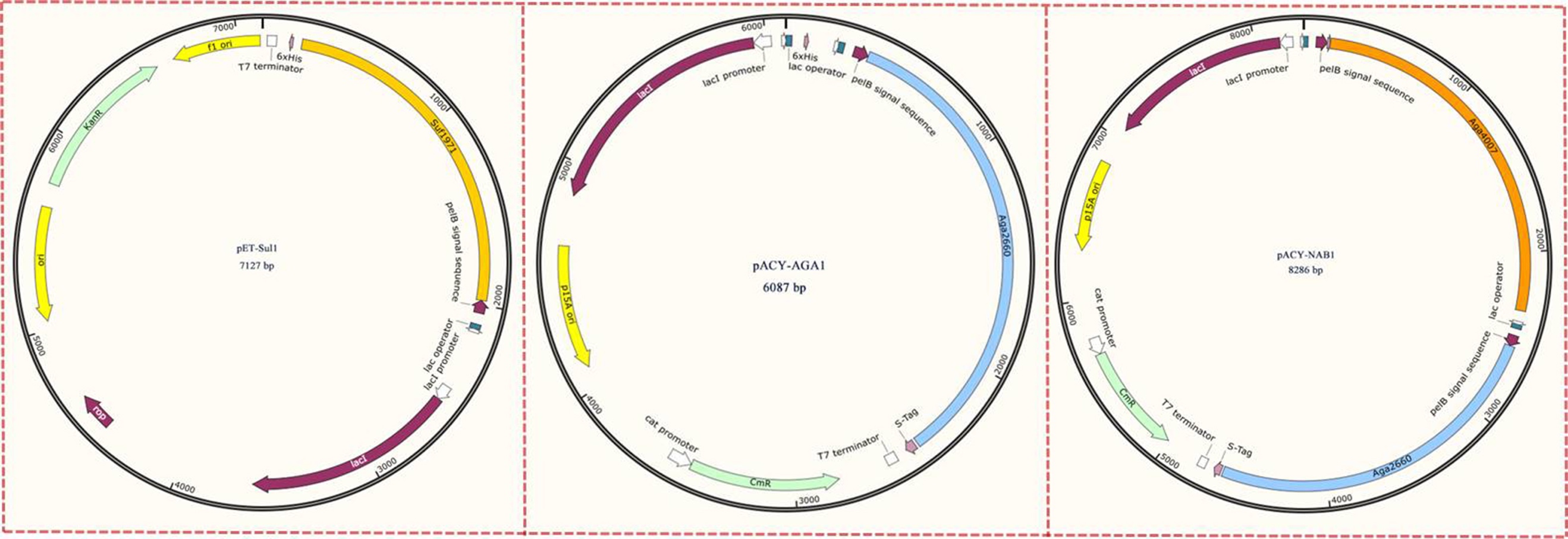
**

**Figure S1.** Plasmids used in the production of agaro-saccharides using engineered *E. coli* BL21(DE3). pET-Sul1 harboring sulfatase Sul1971 was constructed using pET-28a(+), pACY-AGA1 was constructed using pACYCDuet-1, which harbor GH50-dependent Aga2660, plasmid pACY-NAB1 harboring Aga2660 and Aga4007 for the production of NA2.


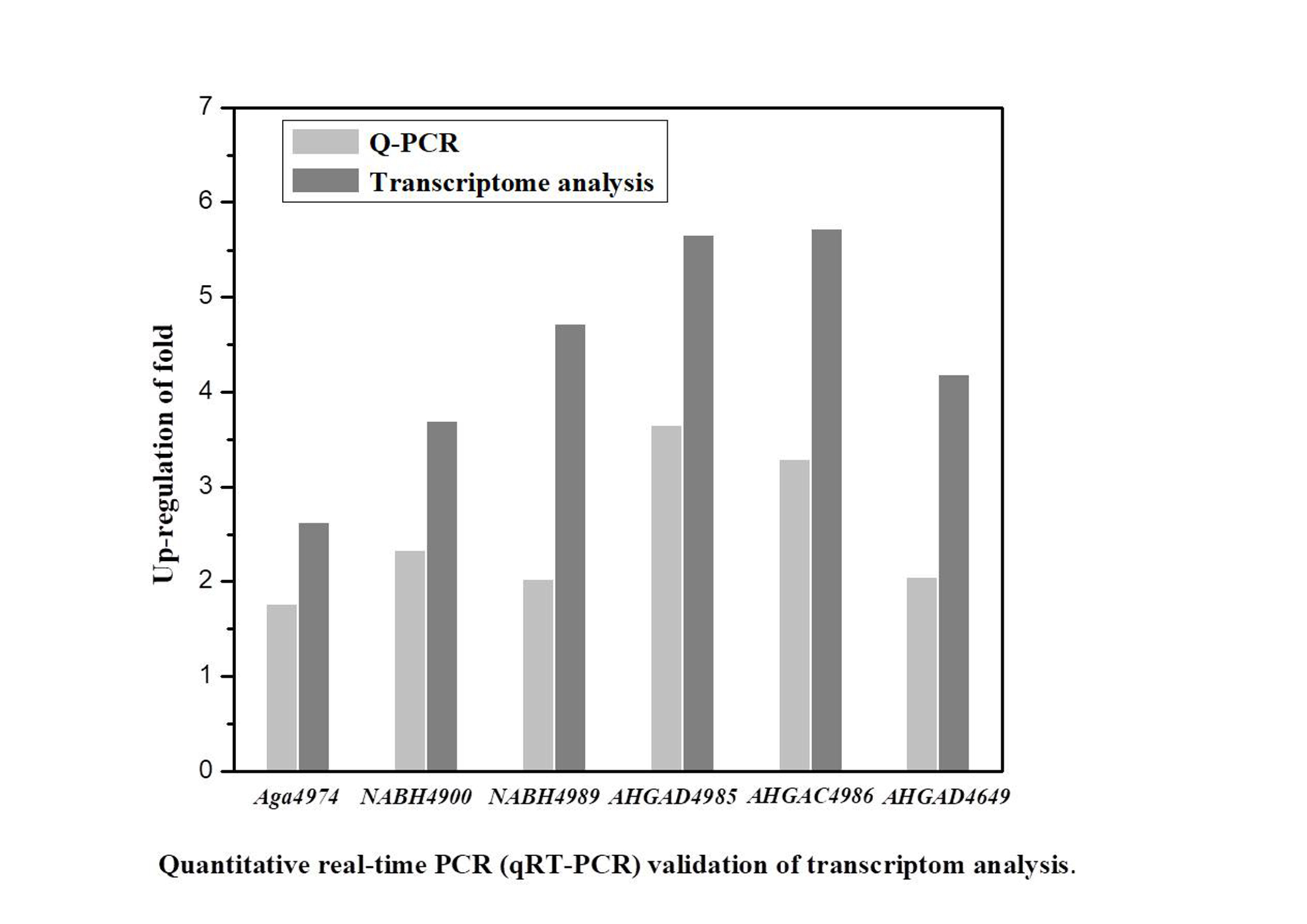


**Figure S2**. Validation of transcriptomic analysis detected the expression level of Aga4974, NABH4900, NABH4989, AHGAD4985, AHGAD4649, and AHGAC4986 by Q-PCR.


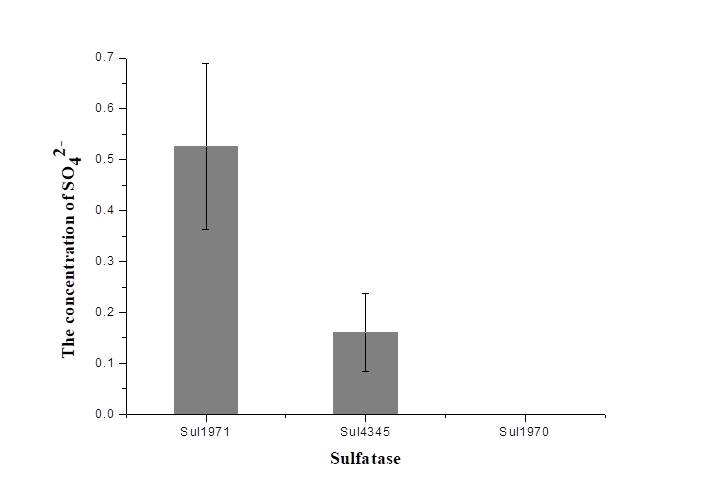


**Figure S3**. Analysis of the concentration of free SO_4_^2−^ ions from the catalysis of crude agarose by sulfatase Sul1971, Sul4345, and Sul1970.


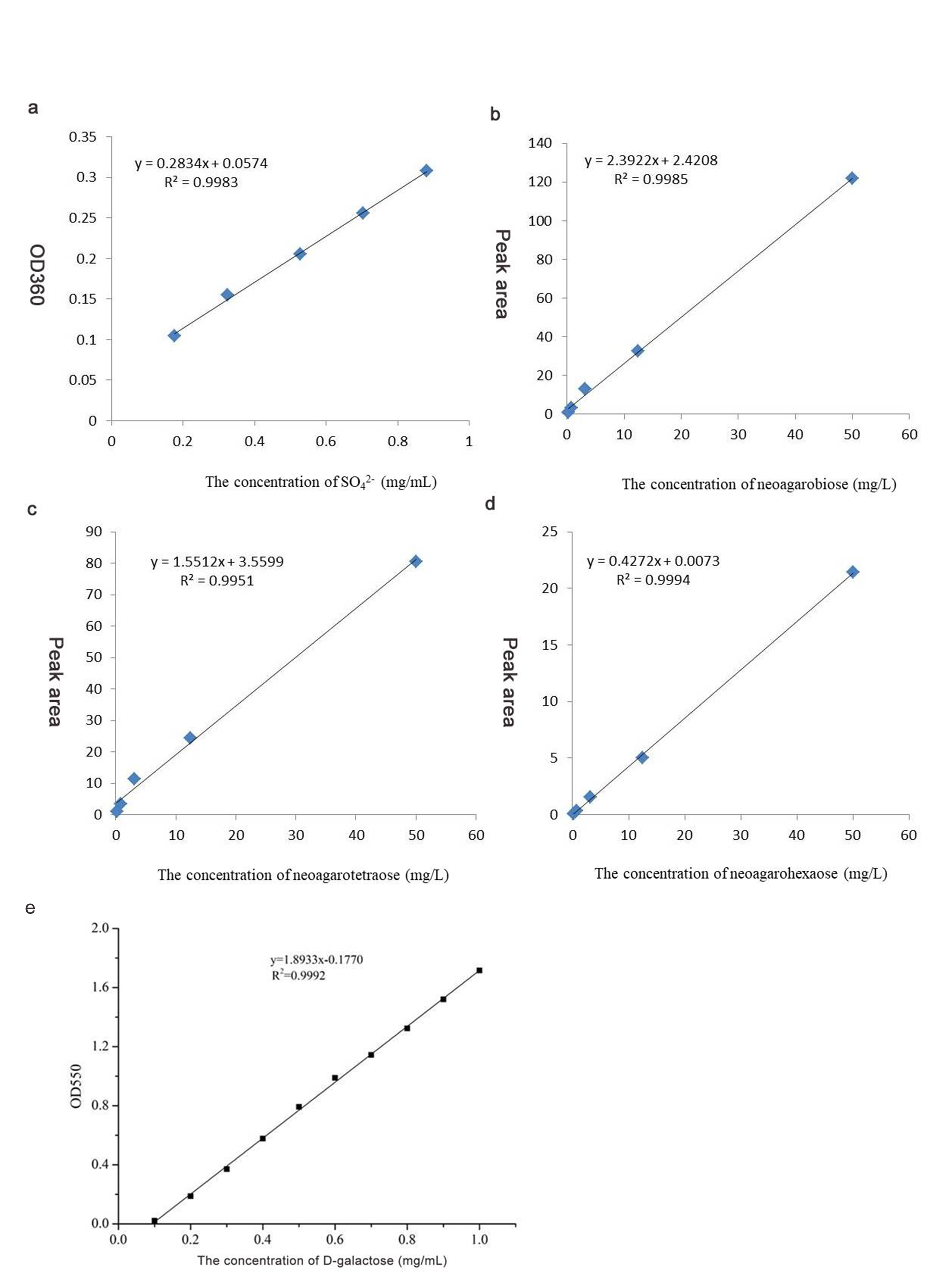


**Figure S4.** Calibration curves of (a) SO_4_^2−^, (b) NA2, (c) NA4, (d) NA6, and (e) reducing sugar

**Supplementary Method**

**Plasmids construction**

The oligonucleotides used in this work are listed in **Supplementary Table 3**. A high-fidelity DNA polymerase kit (Takara, Japan) was used for plasmid construction. pET-Sul1 (**Supplementary Figure 1**) was constructed as follows: Sul1971 gene with *Bam*HI was generated by PCR with primers 1971S-F and 1971S-R from the genomic DNA of the WPAGA1 strain. We added a signal peptide and PelB in the 5ʹ position because the polysaccharide (sulfated agarose) cannot enter the bacterial cell. To add the PelB sequence to the Sul1971 gene, the resulting fragment and oligonucleotide PelB3 were used as templates to perform OE–PCR method using primers 1971S-F and PelB3-R. Plasmid pET28a (+) and resulting fragment were digested with *Bam*HI and *Nco*I after gel purification. Afterward, two fragments were ligated using T4 DNA ligase to generate plasmid pET-Sul1.

Plasmid pACY-AGA1 was constructed by amplifying the Aga2660 gene with primers 2660S-F and 2660S-R from genomic DNA of strain WPAGA1. The resulting fragment and oligonucleotide PelB2 were used as templates by using OE-PCR method with primers PelB2-F and 2660S-R to add the PelB sequence in the 5ʹ position of Aga2660. After digestion with *Nde*I and *Xho*I, the resulting fragment and pACYCDuet-1 were purified by gel purification, and then the Aga2660 gene with PelB was cloned into pACYCDuet-1 to generate the pACY-AGA1 plasmid.

For the construction of pACY-NAB1 plasmid, the Aga4007 gene was amplified by 4007S-F and 4007S-R primers from the genomic DNA of the WPAGA1 strain. The Aga4007 gene fragment and oligonucleotide PelB1 were used as templates to perform OE–PCR method using PelB1-F and 4007S-R primers to add PelB sequence in the 5′ position of Aga4007 gene. The resulting fragment and pACY-AGA1 plasmid were digested by *Nco*I and *Not*I and purified by gel purification. Then pACY-NAB1 was assembled using T4 DNA ligase.

**Preparation of products from enzymatic hydrolysates**

1) *Preparation of products from the enzymatic hydrolysis of agarose*. The enzymatic hydrolysates of five β-agarases and Aga2660 were collected and precipitated with threefold of ethanol. Subsequently, these mixtures were centrifuged at 10,000 × g for 15 min. The supernatants were collected and dried using a rotary vacuum evaporator at 37 °C for 2 h. Three repetitions were performed and finally concentrated fivefold. Then agaro-oligosaccharides were filtered through a membrane filter (pore size, 0.45 µm; Millipore, USA) for TLC plates and ion chromatography analysis.

2) *Purification of AHG and galactose from the enzymatic hydrolysis of NA2*. The corresponding hydrolysates of Aga4007, Aga2660, NABH4454, AHGAD4986, and AHGAC4985 were precipitated with threefold ethanol and were centrifuged at 10,000 × g for 15 min. These supernatants were collected and dried using a rotary vacuum evaporator at 37 °C for 2 h, three repetitions were performed, and finally concentrated fivefold. Subsequently, the concentrated products were loaded onto a Sephadex G-10 column for the removal of the salts. Water was used as mobile phase, and only the sample fractions containing agaro-saccharides, galactose, AHG, AHGA, and KDGal were collected for TLC plate and MS analysis.

3) *Preparation of agaro-saccharides from the production by engineered E. coli*. Ferment liquor (1 mL) was obtained after fermentation for 2, 4, 6, 8, 10, 12, 14, 18, 24, 26, 28, 31, and 48 h using *engineered E. coli*. Then, these samples were centrifuged at 10,000 × g for 10 min, and the supernatants were collected, precipitated with threefold ethanol, and centrifuged at 10,000 × g for 15 min. These supernatants were collected and dried using a rotary vacuum evaporator at 37 °C for 2 h, and three repetitions were performed, and finally redissolved using 1 mL of water for ion chromatography analysis.

4) *Preparation of crude agarose from Gracilaria lemaneiformis.* Dry powder of *Gracilaria lemaneiformis* (5 g) was incubated in 500 mL of water at 80 °C for 4 h. Then the mixture was filtrated using eight layers of gauze, and the filtrate was collected and stored at 4 °C.
